# Supplementary figures and images for: lhCLIP reveals the in vivo RNA–RNA interactions recognized by hnRNPK
Source: PLoS Genet. 2023 Oct 18;19(10):e1011006. doi: 10.1371/journal.pgen.1011006 (PMC10635571; doi:10.1371/journal.pgen.1011006)

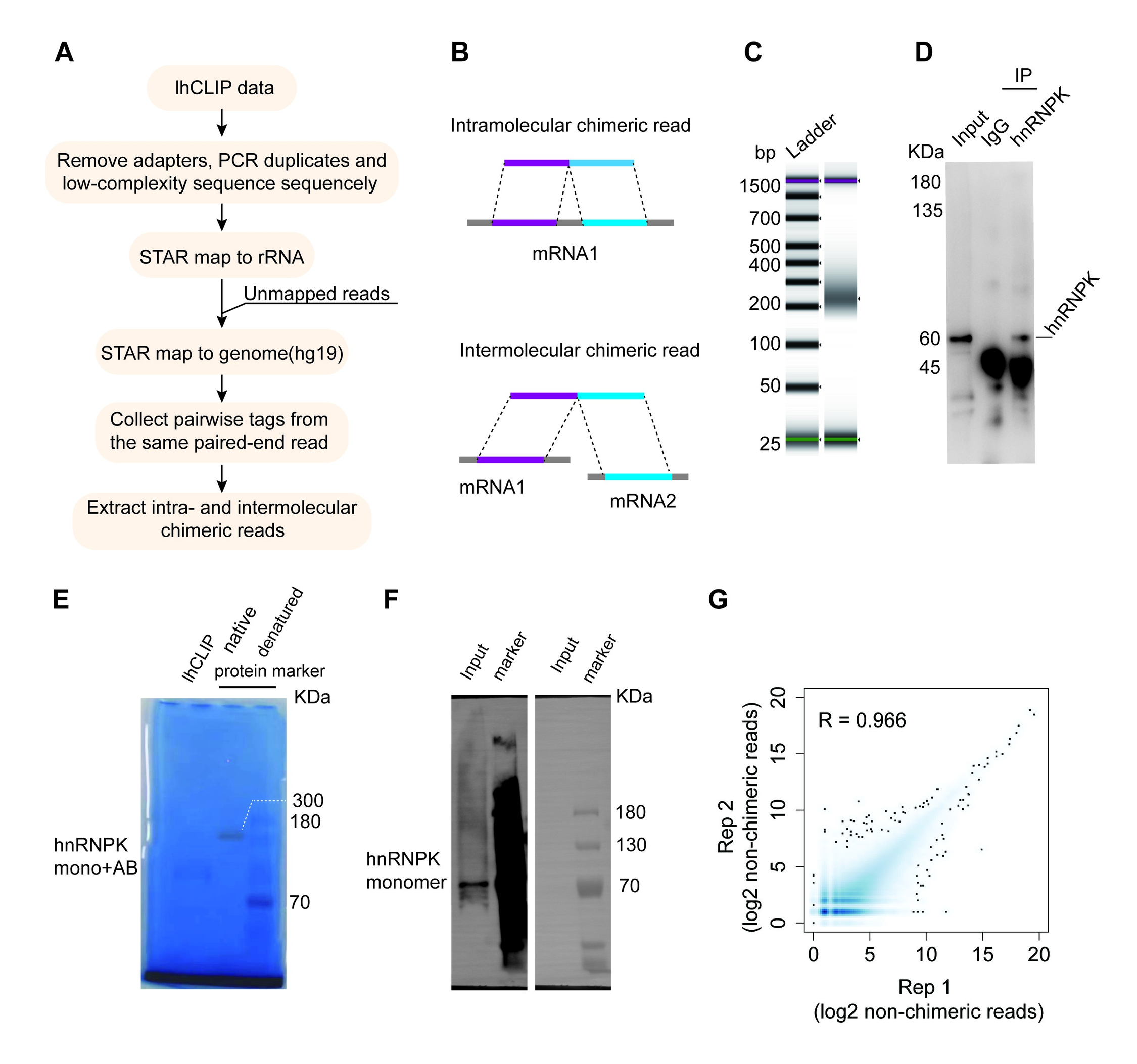

Supplement: S1 Fig — (A) The mapping pipeline for lhCLIP data. (B) The relationship of intra- and intermolecular chimeric reads to reference transcripts. (C) The lhCLIP libraries were quantified using a 2100 Bioanalyzer. (D) Western blot detected the immunoprecipitated hnRNPK. (E) Analysis of the hnRNPK monomer and dimer captured in IhCLIP by blue native gel electrophoresis. The gel image illustrates the Coomassie Blue G-250 stained complex of the hnRNPK monomer along with its antibody. It also displays a native protein mark at 300 KDa, alongside a visible denatured pre-stained protein marker ladder. (F) Analysis of IhCLIP input sample by blue native gel electrophoresis and blotting with anti-hnRNPK antibody. In the left panel, the bands developed through ECL are displayed, while the right panel exhibits a denatured protein marker ladder captured under white light. Notably, the hnRNPK monomer band is evident around 60 KDa. (G) Correlation analysis of the non-chimeric reads between the replicates of lhCLIP. (TIF) [file pgen.1011006.s001.tif]

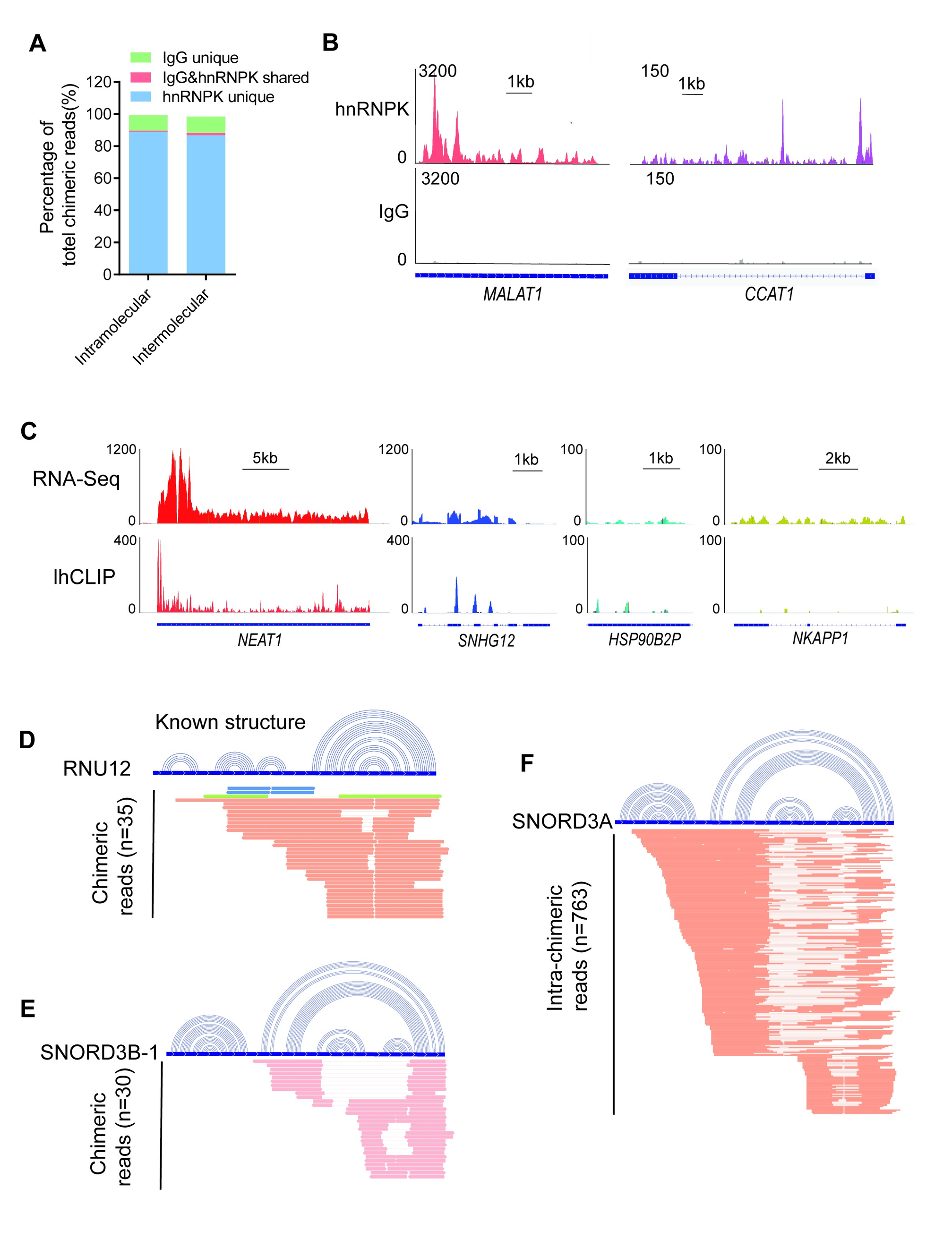

Supplement: S2 Fig — (A) The percentage of chimeric reads that are shared between the IgG and hnRNPK samples. (B) hnRNPK binding sites at MALAT1 and CCAT1 RNA revealed by lhCLIP. (C) Comparing the binding signals of hnRNPK with ncRNAs expressed at different levels. Up, The IGV shows the NEAT1, SNHG12, HSP90B2P, and NKAPP1 RNA-seq reads. Bottom, The IGV shows the NEAT1, SNHG12, HSP90B2P, and NKAPP1 lhCLIP non-chimeric reads. (D), (E) and (F) Comparison of intramolecular interactions of RNU12 (D), SNORD3B-1 (E) and SNORD3A (F) identified by lhCLIP to their know structure. (TIF) [file pgen.1011006.s002.tif]

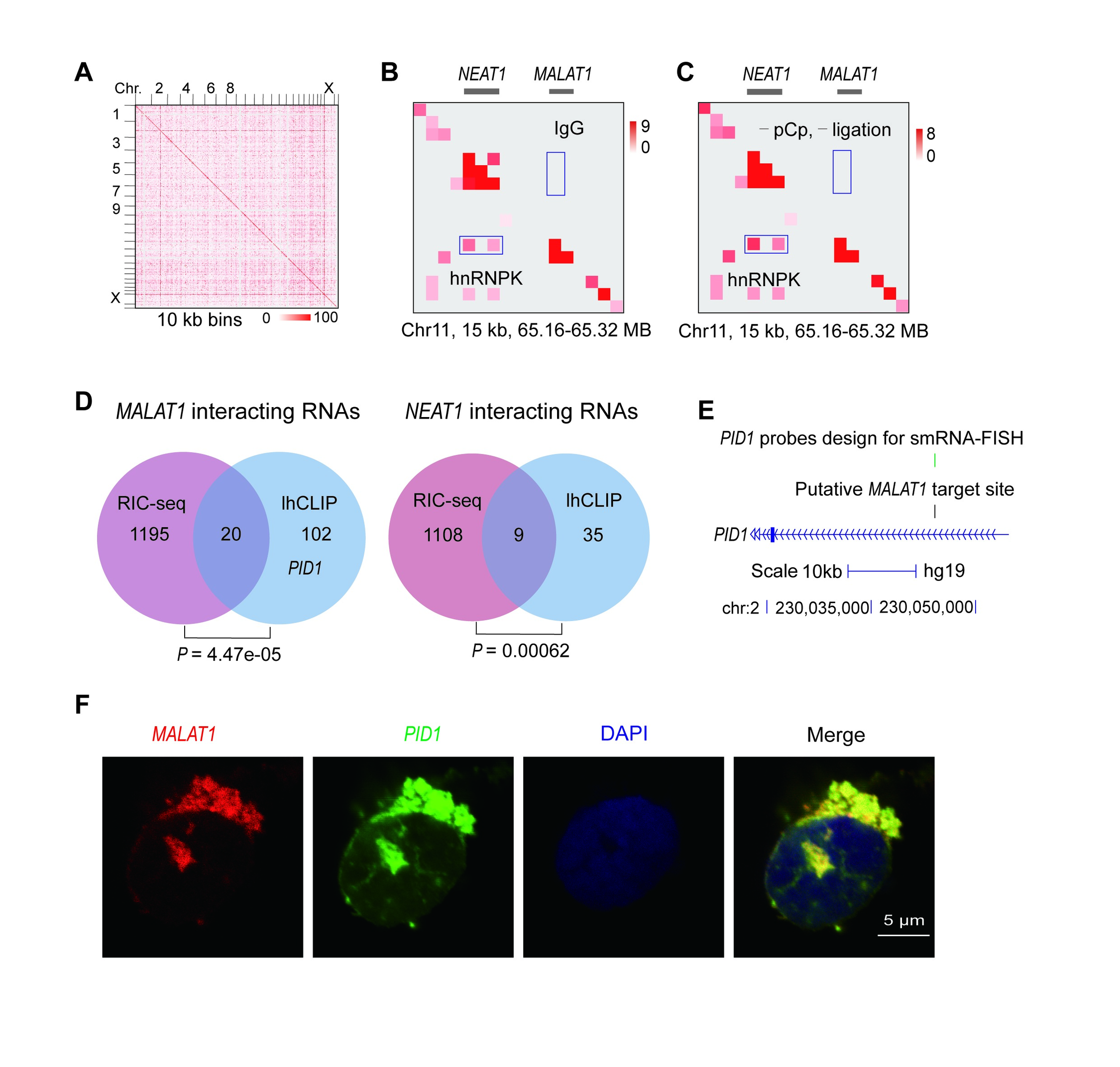

Supplement: S3 Fig — (A) RNA 3D map showing RNA-RNA interactions across all chromosomes. (B) No NEAT1-MALAT1 interaction was detected from IgG sample. hnRNPK sample is shown at the bottom left of the Fig. IgG sample is shown at the top right of the Fig. The blue boxes denote the position of NEAT1-MALAT1 interactions (C) No NEAT1-MALAT1 interaction was detected from control in which pCp-biotin labeling and ligation was omitted. hnRNPK sample is shown at the bottom left of the Fig. Control sample is shown at the top right of the Fig. The blue boxes denote the position of NEAT1-MALAT1 interactions (D) Overlaps of MALAT1 and NEAT1 interacting sites identified by lhCLIP and RIC-seq. Hypergeometric test was used to calculate the P value. (E) Positions of hybridization PID1 probes labelled with digoxigenin. (F) The colocalization of MALAT1 and PID1 pre-mRNA in HeLa cells was detected by smFISH. (TIF) [file pgen.1011006.s003.tif]

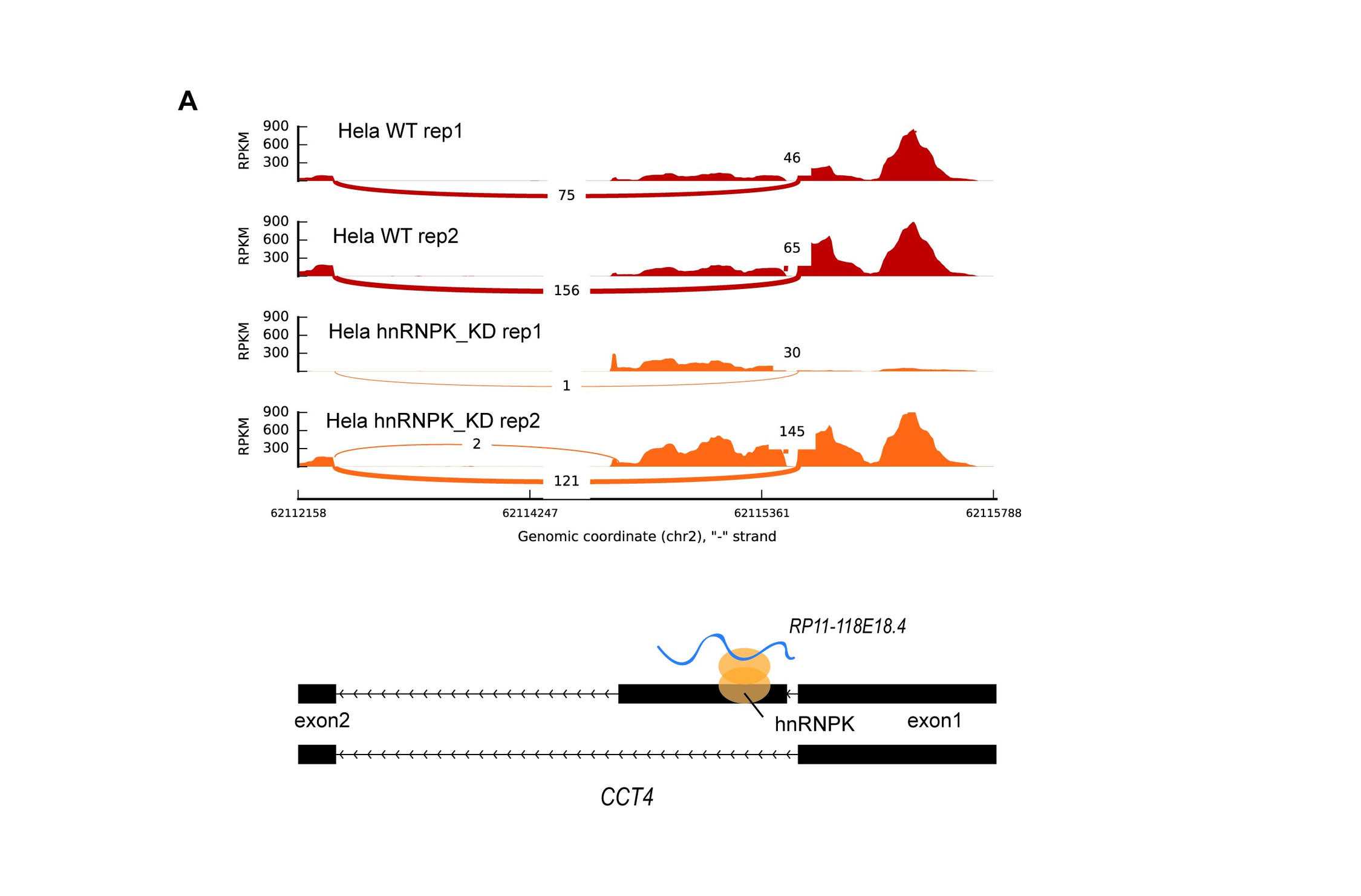

Supplement: S4 Fig — (A) lncRNA RP11-118E18.4 binds to the RNA alternative splicing region of CCT4. Top, Sashimi plots illustrating alternative splicing event of CCT4 in WT HeLa cells and hnRNPK knock down cells. Bottom, Schematic representations of RP11-118E18.4—CCT4 interaction in the RNA alternative splicing region mediated by hnRNPK. (TIF) [file pgen.1011006.s004.tif]
