# Supplementary material for: lhCLIP reveals the in vivo RNA–RNA interactions recognized by hnRNPK
Source: PLoS Genet. 2023 Oct 18;19(10):e1011006. doi: 10.1371/journal.pgen.1011006 (PMC10635571; doi:10.1371/journal.pgen.1011006)
Supplement: S1 Table — (DOCX) [file pgen.1011006.s005.docx]

**Supplementary Table 1**

**The difference between lhCLIP and CRIC-seq**

| Experimental procedure | lhCLIP | CRIC-seq |
| --- | --- | --- |
| Crosslinking method | UV | Formaldehyde |
| pCp-biotin labeling and proximity ligation | On beads | In cells |
| Visualization of RBP-RNA complexes | Chemiluminescent analysis | NO |
| pCp-biotin labelled RNA enrichment | Perform visualization on a nitrocellulose membrane and cut out the colored section | By streptavidin beads |
| The overall complexity of the experimental procedure. | Relatively succinct, suitable for beginners with limited experience | More difficult |
