# Supplementary material for: lhCLIP reveals the in vivo RNA–RNA interactions recognized by hnRNPK
Source: PLoS Genet. 2023 Oct 18;19(10):e1011006. doi: 10.1371/journal.pgen.1011006 (PMC10635571; doi:10.1371/journal.pgen.1011006)
Supplement: S2 Table — (DOCX) [file pgen.1011006.s006.docx]

**Supplementary Table 2**

**Comparative analysis of various techniques for mapping RNA interactions mediated by a specific RBP**

| Method | Crosslinking methods | visualization of protein−RNA  complexes | Chimeric RNA enrichment | Ligation condition | Percentage of chimeric reads | Library time |
| --- | --- | --- | --- | --- | --- | --- |
| CLASH | UV | Radioisotopes | Immunoprecipitation | On beads | ~2% | ~100 h |
| irCLASH | UV | infrared imaging | Immunoprecipitation | On beads | ~7% | ~40 h |
| hiCLIP | UV | Radioisotopes | Immunoprecipitation | On beads | ~2% | ~100 h |
| CRIC-seq | Formaldehyde | NO | Immunoprecipitation and biotin pull-down | In Cell | ~4% | ~90 h |
| lhCLIP | UV | Chemiluminescent analysis | Immunoprecipitation | On beads | ~5% | ~80 h |
