# Supplementary material for: lhCLIP reveals the in vivo RNA–RNA interactions recognized by hnRNPK
Source: PLoS Genet. 2023 Oct 18;19(10):e1011006. doi: 10.1371/journal.pgen.1011006 (PMC10635571; doi:10.1371/journal.pgen.1011006)
Supplement: Attachment — Submitted filename: Response to Reviewers.docx [file pgen.1011006.s012.docx]

Thank you for giving us the opportunity to submit a revised draft of the manuscript “lhCLIP reveals the in vivo RNA–RNA interactions recognized by hnRNPK” for publication in the Journal of PLoS Genetics. We have carefully reviewed the comments and have revised the manuscript accordingly. Our responses are given in a point-by-point manner below and highlighted in blue. Changes to the manuscript are shown in red.

Reviewer #1: Hu et al. have established lhCLIP technology to analyze the RNA-RNA interactions mediated by a specific RNA binding protein (RBP), which provides a useful approach for investigating the roles of specific RBP in RNA metabolism. This approach shows its advances in omitting the application of radioisotopes and detecting only the direct interactions of RNA to specific RBP (crosslinked by UV light). However, two small points need to be clarified to improve the manuscript for publication in PLoS Genetics.

1. The authors claimed that dimerization of HNRNPK could explain the intermolecular RNA-RNA interactions mediated by hnRNPK. However, the co-IP result (Fig S1C) showed that dimerized HNRNPK are barely co-precipitated with the procedure. If the WB was run under denatured condition, the authors at least ran a gel with native condition (similar in Fig 1B) to show that the dimer of hnRNPK was indeed captured in IhCLIP.

Thank you very much for your comments. We used a Blue-native PAGE gel to run the hnRNPK-RNA complex captured in lhCLIP. The lhCLIP sample processing involved UV crosslinking, immunoprecipitation, pCp-biotin labeling, ligation, and elution from beads using the elution/loading master mix (23 µL wash buffer (20 mM Tris-HCl pH 7.4, 10 mM MgCl2, 0.2% Tween-20) and 7.5 µL of 4 × NuPAGE sample buffer without reducing agents), followed by heating at 70 °C for 10 minutes. Subsequently, the sample was transferred from the gel onto a PVDF membrane. After blocking, we incubated the membranes with the primary antibody against hnRNPK and then with horseradish peroxidase-conjugated secondary antibodies. The dimerized hnRNPK band was clearly visualized, as shown in the Figure 1C in the revised article. It's worth noting that both hnRNPK monomer and dimer require heating to eluate from the beads in the lhCLIP process. As a result, their electrophoretic behavior closely resembles that of denatured proteins/marker rather than the native marker. Moreover, it appears that the anti-hnRNPK antibody was co-eluted from the beads along with the hnRNPK protein and could not be separated during blue native gel electrophoresis. This led to the manifestation of a band at approximately 100 KDa for the hnRNPK monomer and its antibody complex. Similarly, the hnRNPK dimer, along with its antibody, was evident as a band around 160 KDa (Figure 1C and Supplementary Figure S1E). In contrast, the input sample, which was only UV cross-linked and then treated with loading buffer, showed a hnRNPK monomer band at around 60 KDa (Figure 1C and Supplementary Figure S1F). The hnRNPK dimer exhibited relatively low abundance, which likely accounts for its undetectable presence in the input sample.

2. Why did the authors not detect the interaction of TERC with RABGGTB using smFISH? Or using RAP-qPCR for PID1 with MALAT1 to confirm the interaction?

Thank you for your valuable suggestions. In the revised version of the article, we have confirmed the interaction of TERC with RABGGTB using smFISH (Figure 3E in revised article) and verified the interaction of PID1 with MALAT1 using RAP-qPCR (Figure 3F in revised article).

Reviewer #2: In this manuscript, the authors developed a novel method for capturing RNA-RNA interactions mediated by a specific RBP, named as lhCLIP. It’s useful and helpful for other researchers, but there are some questions should be addressed before the manuscript can be published:

1. The authors said that the structure of ilCLIP library is the same as that of RIC-seq, while the authors of RIC-seq developed a CRIC-seq (https://doi.org/10.1016/j.molcel.2023.03.001, published in 2023) depending on RIC-seq, and CRIC-seq can capture RNA-RNA interactions mediated by a specific RBP, so the authors should present the difference between ilCLIP and CRIC-seq.

The difference between lhCLIP and CRIC-seq was presented in the following table （We put the table as supplementary Table 1 in revised article）.

**Table R1: the difference between lhCLIP and CRIC-seq**

| Experimental procedure | lhCLIP | CRIC-seq |
| --- | --- | --- |
| Crosslinking method | UV | Formaldehyde |
| pCp-biotin labeling and proximity ligation | On beads | In cells |
| Visualization of RBP-RNA complexes | Chemiluminescent analysis | NO |
| pCp-biotin labelled RNA enrichment | Visualize on a nitrocellulose membrane and excise the colored section. | By streptavidin beads |
| The overall complexity of the experimental procedure. | Relatively succinct, suitable for beginners with limited experience | More difficult |

2. While ilCLIP is a new method, the authors should compare ilCLIP with the other similar methods (CLASH, hiCLIP, irCLASH, CRIC-seq, and so on) in detail, for example, the library time, the chimeric rate, and so on.

The comparison was displayed in the table below（We put the table as supplementary Table 2 in revised article）.

**Table: Comparative analysis of various techniques for mapping RNA interactions mediated by a specific RBP**

| Method | Crosslinking methods | visualization of protein−RNA  complexes | Chimeric RNA enrichment | Ligation condition | Percentage of chimeric reads | Library time |
| --- | --- | --- | --- | --- | --- | --- |
| CLASH | UV | Radioisotopes | Immunoprecipitation | On beads | ~2% | ~100 h |
| irCLASH | UV | infrared imaging | Immunoprecipitation | On beads | ~7% | ~40 h |
| hiCLIP | UV | Radioisotopes | Immunoprecipitation | On beads | ~2% | ~100 h |
| CRIC-seq | Formaldehyde | NO | Immunoprecipitation and biotin pull-down | In Cell | ~4% | ~90 h |
| lhCLIP | UV | Chemiluminescent analysis | Immunoprecipitation | On beads | ~5% | ~80 h |

3. In the manuscript, the authors said that both CLASH and hiCLIP require overexpression of RBP, how can the authors get this conclusion? Sometimes, while there is no effective antibody of target RBP, or the expression of target RBP is low in target cell line, maybe some other causes, the author may overexpress the target RBP with FLAG or GFP label and use the label’s antibody to capture the target RBP’s RNA substrates, but that does not mean that both these methods require overexpression. At the same time, the authors said lhCLIP does not require the introduction of any exogenous protein-coding genes, so while there is not the target RBP’s antibody, how can you using lhCLIP to capture the target RBP’s RNA substrates?

The expression levels of AGO1 (~3 RPKM) and STAU1 (~20 RPKM), which are target RBPs in CLASH (PMID: 23622248) and hiCLIP, are not considered low in human cells. Moreover, antibodies for these RBPs are available in the market. In both CLASH and hiCLIP, the percentage of chimeric reads is only about 2%. Therefore, to obtain a sufficient number of chimeric reads for subsequent analysis, it is necessary to overexpress these RBPs. You are correct. If there is no effective antibody available for the target RBP or if the expression of the target RBP is low in the specific cell line, it becomes necessary to overexpress the target RBP with a FLAG or GFP label and use the label’s antibody to capture the target RBP’s RNA substrates in lhCLIP.

We emphasis the sensitivity of lhCLIP in capturing endogenous expressed proteins which is not presented by CLASH or hiCLIP method. However, in order to avoid any misunderstanding, we edited the related description in the revised article (line 103-108).

4. The authors should present the detailed messages for their analysis, for example, how to annotate the chimeric or non-chimeric reads, how to define the cluster in Figure 2C, and so on; the detailed parameters for each software; for some figures, the authors should write the figure legend in detail, for example, the Figure1B.

Thank you for your valuable suggestions. In the revised version of the article, we have provided detailed explanations for lhCLIP data processing and analysis. These explanations cover topics such as annotating chimeric or non-chimeric reads (elucidated in lines 569-590), defining clusters (in lines 586-588) in Figure 2C, and specifying parameters for each software used (in lines 561-590). To further clarify, intra-molecular chimeric reads with different termini but overlapping at both arms can be classified as high-confidence interactions if the connection score exceeds 0.01. For instance, as shown in Figure 2D, all intra-chimeric reads form a single cluster. We have also provided a detailed figure legend in the revised version of the article. For Figure 1B, we meticulously elaborated on the details of individual sample treatments within the figure legend （in lines 857-860）. For Figure 2, we reorganized the sequence of Figure 2D-G to enhance the clarity of the presented results（in lines 877-885）. For Figure 2D, we described in detail the information of Intra-chimeric reads in red box in the figure legend (in lines 879-880). We added “The orange oval represents the hnRNPK protein” in the figure legend of Figure 4 (in line 908, 912-913).

5. There are some wrong spelling, for example, in Figure 3A, should be “contacts”, in line 193, should be “number”, and so on.

Thank you very much for pointing out these errors. We have thoroughly reviewed the entire article and corrected the spelling mistake including the mentioned ones.

Reviewer #3: The manuscript “IhCLIP reveals the in vivo RNA-RNA interactions recognized by hnRNPK”, Hu et al., developed IhCLIP that can investigate RNA-RNA interactions and the mediated specific proteins with UV crosslinking and proximity ligation and immunoprecipitation techniques. Big advantage of the method is that it provides both information of RNA-RNA and mediated specific protein complex at same time. I believe that the IhCLIP is important for further interactome analysis study, however I have several comments that authors should address.

In this study, authors selected hnRNPK to investigate their IhCLIP technology. hnRNPK is one of most famous RNA binding proteins that is known to bind many types of RNAs for splicing function, nucleocytoplasmic function and mRNA translation. I wonder whether the technology can be broadly used for other RBPs.

Thank you very much for your comments. lhCLIP can be used for these RBPs that, akin to hnRNPK, function as a homodimer. Examples of such proteins include hnRNPA1 and PTBP1 (PMID: 36958328). In addition, the lhCLIP methodology shares common steps with both irCLASH (PMID: 32203492) and hiCLIP (PMID: 25799984), including the employment of identical UV crosslinking and immunoprecipitation techniques. Therefore, lhCLIP can be applicable for double stranded RNA binding proteins, as hiCLIP and irCLASH have been employed to study RNA-RNA interactions facilitated by these proteins. If the RBP only forms monomers and facilitates RNA-RNA interactions with other proteins, the UV crosslinking step used in lhCLIP should be substituted with formaldehyde crosslinking or formaldehyde plus disuccinimidyl glutarate crosslinking. These alternative crosslinking methods can crosslink protein-RNA and protein-protein interactions, enabling the capture of both direct and indirect RNA-RNA interactions bound by proteins.

In addition, authors focus on very highly expressed nuclear transcripts such as NEAT1, MALAT1, snRNA, snoRNA etc. in this study. I would imagine that capturing and sequencing of hnRNPK binding on such RNAs are much easier than lowly expressed ncRNAs and lncRNAs. Authors must investigate lowly expressed RNA-RNA interactions and mention the limitation of the technology.

We examined RNA-RNA interactions involving lowly expressed RNAs. The data are added in the revised article. Our findings indicate that the binding of hnRNPK to lowly expressed RNAs can be captured by lhCLIP (see the following Figure which also available in Supplementary Figure S2C in the revised article). Furthermore, RNA-RNA interactions of these lowly expressed RNAs can also be captured by lhCLIP, such as the interaction between *NKAPP1* and *TMEM255A* (Supplementary table 6). The following sections provide a more detailed description of the examination process: Initially, we assessed the expression of RNAs in HeLa cell using RNA-seq data and selected three relatively lowly expressed ncRNAs: *SNHG12*, *HSP90B2P*, and *NKAPP1*. Subsequently, we investigated hnRNPK binding signals for these RNAs, which are non-chimeric reads captured by lhCLIP. Our findings indicate that the detected binding sites are less and the read amounts of each site are lower on the lowly expressed RNAs compared to the highly expressed *NEAT1*, as depicted in the following figure (also in Supplementary Figure S2C in the revised article). Thus, the binding of hnRNPK to RNA is determined not only by the specific RNA sequence, but also the abundancy of the RNA. Lastly, we compiled a list detailing the inter-molecular RNA-RNA interactions involving the prominently expressed *NEAT1* transcript, alongside the lowly expressed transcripts *SNHG12*, *HSP90B2P*, and *NKAPP1*.  Even though *NEAT1* interacts with a wide variety of diverse transcripts, it is surprising to discover that the minimally expressed *NKAPP1* demonstrates a contact count of 9 with *TMEM255A*, surpassing the majority of contact counts (ranging from 2 to 10) between NEAT1 and its interacting transcripts (Supplementary Table 6 in revised article). This observation indicated that lhCLIP can detected RNA-RNA interactions of low-expressed RNAs. Nevertheless, we discussed the limitation of the technology in lines 345-354 of the revised article.

The advantage of this method is that the RNA-RNA interactions are captured after crosslinking and proximity ligation. So that we can capture many RNAs that are not only directly bind hnRNPK but also bind hnRNPK binding proteins. However, I am afraid if we can understand whole interacted molecules of such splicing complexes with this technology because other proteins than hnRNPK are missing from the analysis. Authors should discuss and suggest further possible analysis or validation of interactome study with the technology.

The UV crosslinking used in lhCLIP only forms covalent bonds at sites of direct protein–RNA interactions thereby most of the indirect RNA-RNA interactions that mediated by hnRNPK and other proteins are missing from the analysis. To gain a comprehensive understanding of the interactions among molecules in splicing complexes, the initial approach involves substituting the UV crosslinking method with formaldehyde crosslinking, which crosslinks protein-RNA and protein-protein interactions and thus can capture both indirect interactions as well as direct interactions that are caged or flanked by proteins. Alternatively, we can fix cell with both formaldehyde and disuccinimidyl glutarate, a strong protein-protein crosslinker, to more efficiently capture RNAs linked indirectly through multiple protein intermediates. Then, RAP-qPCR/Seq, RAP-WB/MS, and IP-WB/MS are utilized to validate the interactome involving RNA-RNA, RNA-protein, and protein-protein interactions, respectively. We added this contains in discuss part (in lines 345-354).

CLIP technologies are depending on antibody affinity to target protein to capture protein-RNA complexes. Even though we select two different types of antibodies to same target protein, results can be different with different antibodies. Can authors suggest how we can select good and proper antibodies for the specific results of IhCLIP and how we can understand that the results are not false positive?

It is true that the quality of the antibodies is a key factor for success lhCLIP. In our practice,

we selected antibodies that have been extensively employed in iCLIP/eCLIP studies with numerous published papers. If the convinced antibody is not available, as you proposed, choosing more than one type of antibodies and analyze the data by combined or comparison analysis is necessary. To verify true positive results, the following methods we may use. Firstly, comparing the non-chimeric reads of lhCLIP with traditional CLIP reads when using the same antibody; secondly, confirming the similarity of RNA secondary structures obtained through lhCLIP with known structures; and finally, validating inter-chimeric reads indicating interactions between two distinct RNAs using smFISH, RAP-qPCR and other experimental methods.
